# Cytological aspects of pollen germination in *Mauritia flexuosa* (Arecaceae)

**DOI:** 10.1007/s00709-026-02161-6

**Published:** 2026-02-05

**Authors:** Maria Júlia Gomes Marques, Leonardo Monteiro Ribeiro, Rúbia Santos Fonseca, Hellen Cássia Mazzottini-dos-Santos

**Affiliations:** 1 Laboratório de Anatomia Vegetal, Programa de Pós-Graduação em Botânica Aplicada, Universidade Estadual de Montes Claros, 39401-089 Montes Claros, Brazil; 2 Programa de Pós-graduação em Produção Vegetal, Instituto de Ciências Agrárias, Universidade Federal de Minas Gerais, Montes Claros, 39404-006 Brazil; 3 Laboratório de Reprodução Vegetal, Departamento de Biologia Geral, Universidade Estadual de Montes Claros, Montes Claros, 39401-089 Brazil

**Keywords:** Male gametes, Palm pollen, Pollen composition, Pollen tube development, Sporoderm ultrastructure

## Abstract

Little is known about the dynamics of pollen germination in palms. *Mauritia flexuosa* (buriti) is native to the Amazon but also occurs in wetland areas (veredas) within the Cerrado. The species is dioecious and, in this environment, exhibits supra-annual flowering, which underscores the importance of post-pollination events for its reproductive success. The aim of this study was to define the stages of buriti pollen germination, to describe the mobilization of reserve compounds, and to examine subcellular changes during pollen tube development. Cytochemical tests and ultrastructural analyses were performed throughout the germination process and the in vitro development of the pollen tube. The pollen of *M. flexuosa* is sphero-oblate and monoporate. The exine is thick and impregnated with structural phenolic compounds, with pointed spicules and pollenkitt. The intine is thin, becoming thicker in the pore region, where it exhibits a mixed composition. The protoplast of the vegetative cell is rich in reserve compounds. Germination occurs in four phases. In the pore region, the pectin-rich intine ensures effective rehydration. The mobilization of starch and lipids, in coordinated stages, provides energy for pollen tube elongation, accompanied by continuous wall synthesis. Protoplast reorganization supports pollen tube growth, with proliferation of dictyosomes, endoplasmic reticulum, mitochondria, and ribosomes. The second mitosis takes place shortly after pollen tube emergence, contributing to rapid fertilization. The structural features of the pollen and the dynamics of germination favor the reproductive success of *M. flexuosa*, particularly in semiarid environments.

## Introduction

The success of pollen germination and pollen tube development is crucial for the reproduction of angiosperms (García et al. 2017; Johnson et al. 2019). These processes are coordinated by a complex signaling network that extends up to fertilization (Zhou et al. 2022). Following pollen recognition by the stigma, a series of subcellular changes occurs, culminating in pollen tube protrusion (Firon et al. 2012; Johnson et al. 2019; Hafidh and Honys 2021; Kim et al. 2021; Robichaux and Wallace 2021). The structure of the sporoderm, particularly the exine composition enriched with sporopollenin, provides desiccation resistan ce and prolongs microgametophyte viability (Ariizumi and Toriyama 2011). In this context, beyond its structural architecture, the chemical composition of the vegetative cell plays a central role in maintaining viability and regulating the growth pattern of the pollen tube, traits that vary significantly among species (Pacini et al. 2006; Hafidh et al. 2016; Shivanna and Tandon 2020). Despite advances in the understanding of pollen germination, significant gaps remain, especially concerning ultrastructural modifications of the vegetative cell protoplast during the early stages of the process.

The mobilization of pollen reserves, together with water content and the secretory activity of the stigma, directly influences the rate of pollen tube growth and fertilization (Lichocka et al. 2018; Rozier et al. 2020; Bordeleau et al. 2022; Cortez et al. 2022). To meet the high energy demand of the elongating tube, storage compounds are mobilized and directed toward the apical region, where cell elongation occurs (Vesprini et al. 2002; Hafidh et al. 2016). Although studies addressing the mechanisms of tube elongation exist, few have examined the patterns of reserve mobilization. In this regard, cytochemical analyses are essential for identifying the storage compounds involved in pollen tube protrusion and growth.

The Arecaceae family exhibits notable peculiarities in its reproductive traits, often closely linked to the environment in which they occur. Among these characteristics, the predominance of bicellular pollen dispersal stands out (Dransfield et al. 2008; Lora et al. 2012). The dynamics of pollen germination influence the reproductive success of species (Berger and Twell 2011; Hafidh et al. 2016). In species with bicellular pollen, the second mitosis that gives rise to the male gametes may occur after germination or within the pollen tube (Lora et al. 2012), but it is most commonly observed at the very onset of tube protrusion (Shivanna and Tandon 2020). However, the timing of this process remains uncertain for the Arecaceae family. Expanding knowledge of the reproductive processes of palms is important to support the development of conservation strategies, particularly in the face of climate changes that threaten the perpetuation and biodiversity of ecosystems.

*Mauritia flexuosa* L.f. (buriti), an Amazonian palm, is widely distributed throughout Tropical America, including the Brazilian Cerrado, where it plays a crucial role in the vegetational formations known as veredas (Lorenzi et al. 2010; Nunes et al. 2022). The species contributes to hydric stability in these environments and provides food and shelter for wildlife (Araújo et al. 2002; Martins et al. 2014; Nunes et al. 2022). Its oily fruits and other products are economically important for traditional communities, with potential for cosmetics and biofuel production (Lorenzi et al. 2010; Dresselhaus and Franklin-Tong 2013; Virapongse et al. 2017). Phenological studies in savanna environments indicate a supra-annual flowering and fruiting pattern in *M. flexuosa*, with peaks during the rainy season (Ávila et al. 2023). The species is dioecious, and flowering synchrony is essential for fruit production (Nadot et al. 2016). Pistillate flowers remain on the rachilla for approximately five days (Rosa and Koptur 2013; Ávila et al. 2023), during which pollen deposition, germination, and pollen tube development must occur to ensure fertilization. The species exhibits long periods without flowering, highlighting the importance of understanding pollen germination and pollen tube growth dynamics, which are indispensable for fertilization. Anthropogenic activities have threatened the conservation of *M. flexuosa* in the Cerrado biome (Araújo et al. 2002; Silva and Scariot 2013), and information about its reproductive cycle can provide valuable insights for domestication and propagation projects.

The aim of this study was to characterize the dynamics and cytological aspects of pollen germination in *M. flexuosa* and to address the following questions: (i) What are the micromorphological, cytochemical, and ultrastructural characteristics of the pollen? (ii) What are the phases of pollen germination, and what is the dynamics of stored compound mobilization and subcellular changes? Additionally, this study sought to relate the pattern of pollen tube development to ecological aspects, considering the semi-arid environments in which the species occurs.

## Materials and methods

### Collection of plant material

Staminate flowers, at pre-anthesis and anthesis stages, were collected from five *M. flexuosa* individuals, at different positions of the inflorescences, during the peak flowering period, in February 2023. Collections were carried out in a natural population located at Vereda Almescla, within the Rio Pandeiros Environmental Protection Area (15°20′54.9″ S, 44°53′84.5″ W), Bonito de Minas municipality, northern Minas Gerais, Brazil. Due to the large size of the sampled individuals, a ladder and long-reach pruning shears were used for flower collection. Pollen was removed from the anthers using a brush and subsequently dried in an air-circulated oven at 30 °C, for 24 h (Novara et al. 2017).

### Pollen micromorphology

For micromorphological analysis, *M. flexuosa* pollen was fixed in Karnovsky’s solution (Karnovsky 1965), dehydrated in an ethanol series, critical-point dried in CO₂, and mounted on aluminum stubs, then coated with a 10 nm layer of gold using a BalTec metalizer (Leica Microsystems, Heidelberg, Germany) (Robards 1978). The material was examined using a Quanta 200 scanning electron microscope (FEI Company, Eindhoven, The Netherlands), and digital images were captured at 12–20 kV.

### Pollen and pollen tube anatomy and cytochemistry

Anthers from staminate flowers at anthesis were fixed in Karnovsky’s solution (Karnovsky 1965), dehydrated in an ethanol series, and subsequently embedded in 2-hydroxyethyl methacrylate (Leica Microsystems, Heidelberg, Germany) (Paiva et al. 2011). Cross sections (5 μm thick) were obtained using a rotary microtome (HistoCore Autocut, Nussloch, Germany). Sections were stained with 0.05% toluidine blue, pH 4.7 (O’Brien et al. 1964; modified), to identify mucilage and phenolic compounds; Lugol’s reagent (Johansen 1940) for starch; xylidine-ponceau (Vidal 1970) for proteins; sudan black (Pearse 1972) for total lipids; nile blue sulfate (Cain 1947) for neutral and acidic lipids; and autofluorescence under UV light for lignified cell walls.

To promote germination, pollen was placed on four Petri’s dishes containing a culture medium composed of 0.005 g/L H₃BO₃, 0.015 g/L Ca(NO₃)₂, 0.010 g/L MgSO₄, 0.005 g/L KNO₃, 0.5 g/L agar, and 5.0 g/L sucrose (Sousa et al. 2010; adapted). The plates were maintained in a BOD germination chamber, at 30 °C, for 2 h, in the absence of light. Germinated pollen was dehydrated through an acetone series and infiltrated with Araldite resin (Leica Microsystems, Heidelberg, Germany) (Robards 1978). Semithin sections were obtained using a UCB ultramicrotome (Leica Microsystems, Heidelberg, Germany) and stained with 0.05% toluidine blue, pH 4.7 (O’Brien et al. 1964; modified). Germinated pollen was also analyzed fresh using the previously described histochemical tests. Images were captured with a photomicroscope (Zeiss Lab AI/Axion Cam ICC 3, Jena, Germany).

### Pollen and pollen tube ultrastructure

Nine pollen samples were collected from the in vitro culture at 15 min intervals over a period of 2 h. Samples were fixed in Karnovsky’s solution (Karnovsky 1965), for 24 h, and post-fixed in 1% osmium tetroxide (in 0.1 M phosphate buffer). The material was dehydrated using an acetone series, infiltrated with Araldite resin (Leica Microsystems, Heidelberg, Germany) (Robards 1978), and ultrathin sections were obtained using a UCB ultramicrotome (Leica Microsystems, Heidelberg, Germany). Contrast staining was performed with uranyl acetate (Watson 1958) and lead citrate (Reynolds 1963). Images were digitally captured using a Tecnai G2-20 SuperTwin transmission electron microscope (FEI Company, Eindhoven, The Netherlands) operating at 200 kV.

## Results

Four distinct phases of *M. flexuosa* pollen germination were identified based on structural and cytological modifications: (I) immature bicellular pollen; (II) initiation of germination, characterized by polarization of the vegetative cell; (III) pollen tube emergence, accompanied by extensive organelle proliferation; and (IV) mature tricellular pollen and pollen tube elongation. Precise temporal assessment of these germination stages is precluded by their pronounced heterogeneity.

**Fig. 1:**
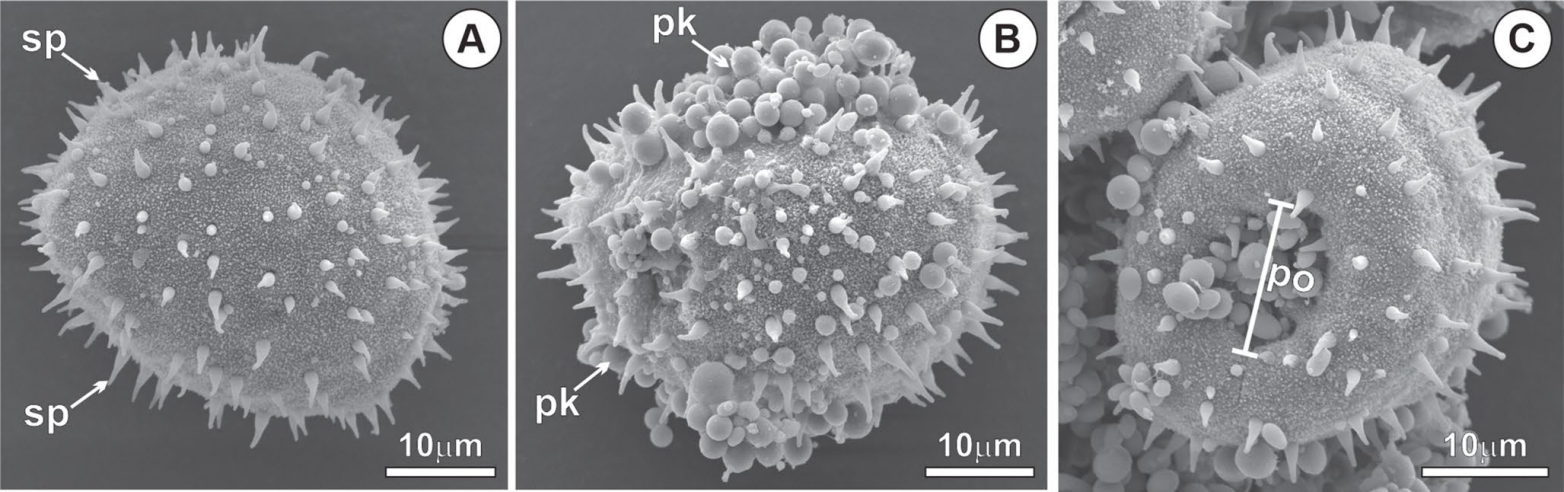
Pollen micromorphology of *Mauritia flexuosa* after anthesis - phase I. (**A**) Spicules on the exine. (**B**) Pollenkitt associated with the sporoderm. (**C**) Pollen pore region. sp, spicules; po, pore; pk, pollenkit

**Fig. 2:**
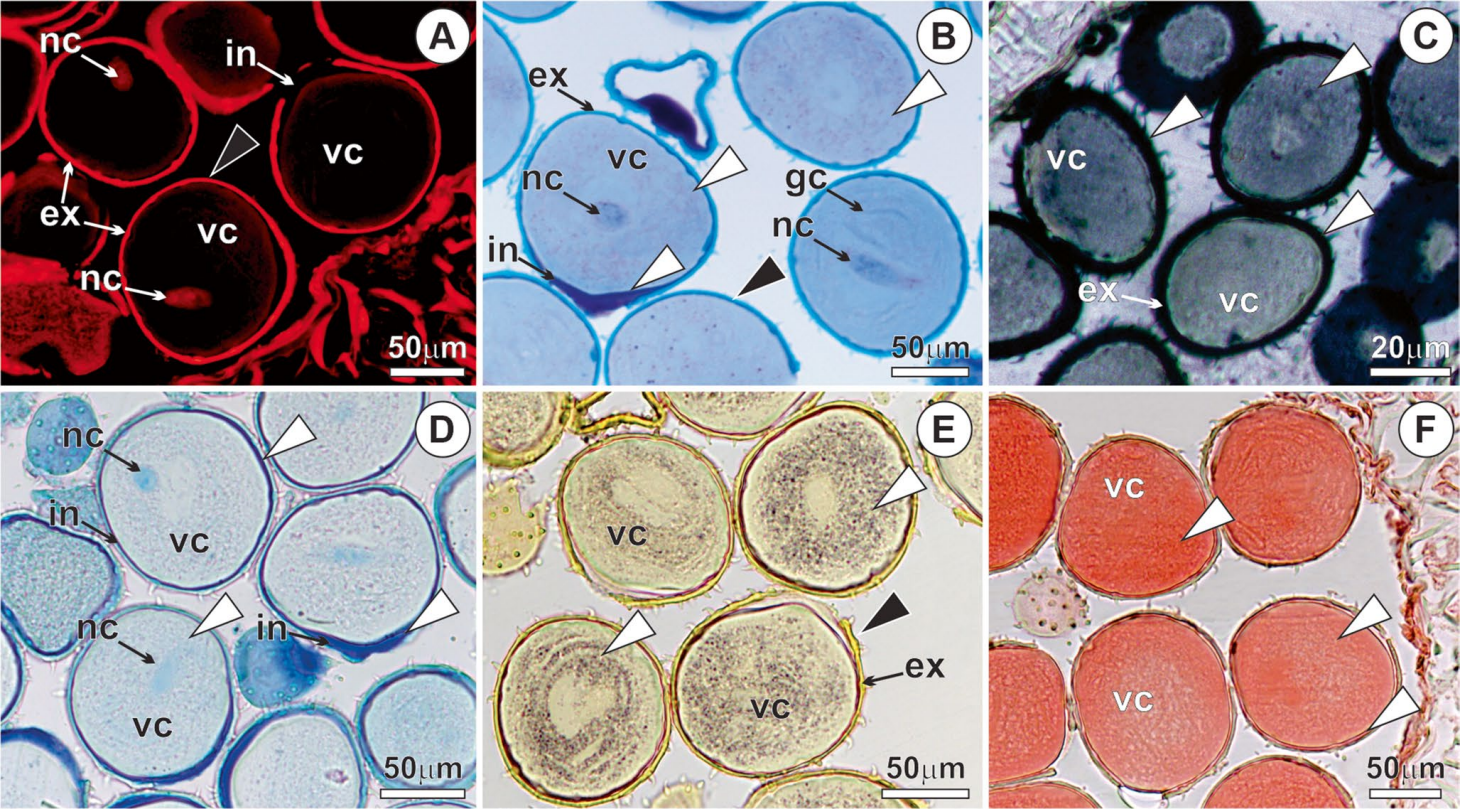
Histochemistry of *Mauritia flexuosa* pollen after anthesis - phase I. (**A**) Phenolics present in the exine (black arrowhead), under autofluorescence with Rhod filter. (**B**) Spicules and exine containing phenolic compounds (black arrowhead; green staining); intine and vegetative cell protoplast rich in mucilage (white arrowheads; purple staining). (**C**) Lipid compounds in the exine, constituents of the pollenkitt, and in the vegetative cell protoplast (white arrowheads), black staining with sudan black. (**D**) Acidic lipid compounds (white arrowheads), blue staining with nile blue. (**H**) Phenolics in the exine (black arrowhead) and starch in the vegetative cell protoplast (white arrowheads), black staining with Lugol’s reagent. (**I**) Proteins in the vegetative cell protoplast (white arrowheads), red staining with xylidine-ponceau. ex, exine; gc, generative cell; in, intine; nc, vegetative cell nucleus; po, pore; pk, pollenkitt; vc, vegetative cell

The pollen of *M. flexuosa* is monoporate or monosulcate, exhibits an oblate-spherical shape, and possesses an exine ornamented with pointed spicules, accompanied by abundant pollenkitt (Fig. 1a-c). In Phase I, the exine is thick and impregnated with structural phenolic compounds (Fig. 2a-b). Lipids are present in the pollenkitt, exine, intine, and the vegetative cell protoplast (Fig. 2c), with acidic lipids predominating in the intine (Fig. 2d). In the pore region, the intine shows conspicuous thickening and mixed composition, with a predominance of pectins (Fig. 2b). The vegetative cell protoplast is rich in reserves, particularly starch (Fig. 2e) and protein (Fig. 2f).

**Fig. 3:**
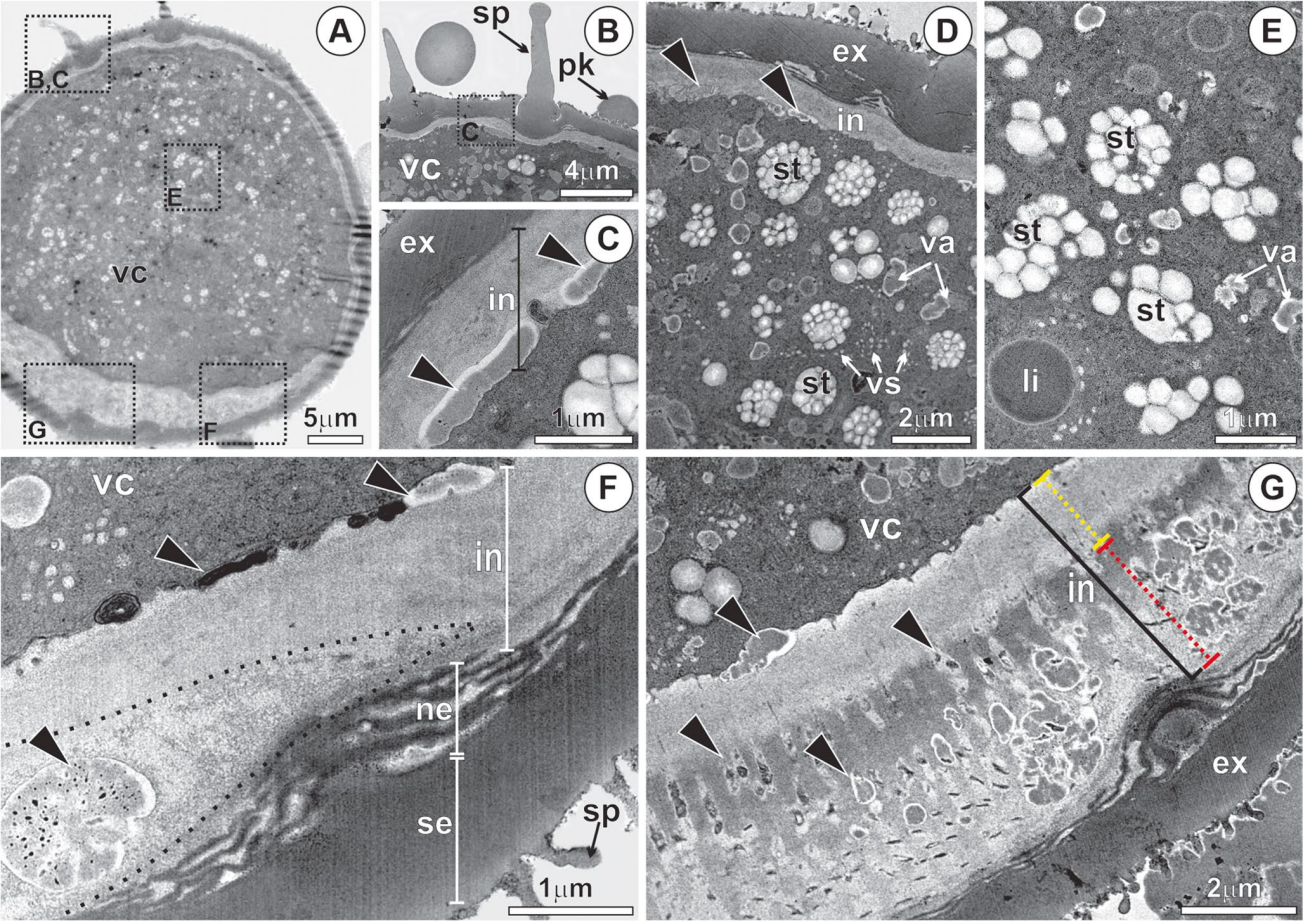
Ultrastructural analysis of *Mauritia flexuosa* pollen - phase I. (**A**) Longitudinal section of the pollen after anthesis, showing its internal structure in preparation for germination. (**B**) Pollenkitt and spicules associated with the sporoderm, which assist in pollen adhesion to the stigma. (**C-D**) Secretion of pectic substances that adhere to the intine (black arrowheads). (**D-E**) Vegetative cell protoplast, with abundant reserves in small vacuoles, amyloplasts and lipid droplets. (**F**-**G**) The intine with two regions: inner (yellow dotted) and outer (red dotted); note the accumulation of pectins (black arrowheads) beneath the inner intine and within the outer intine, leading to an increase in its thickness in the pore region. ex, exine; in, intine; ne, nexine; pk, pollenkitt; se, sexine; sp, spicule; st, starch; va, vacuole; vc, vegetative cell; vs, vesicles

The pollen grain has two distinct regions of the sporoderm, the exine and the intine (Fig. 3a, f-g), and a vegetative cell rich in reserves (Fig. 3a, d-e). The exine is divided into two distinct layers: the sexine, the outer portion, and the nexine, the inner portion (Fig. 3f). The sexine is thicker and more electron-dense, displaying depressions from which the spicules project (Fig. 3a, f-g). The nexine exhibits a looser organizational pattern, consisting of alternating electron-dense and electron-lucent layers (Fig. 3f). In most of the sporoderm, the intine forms a thin, slightly electron-dense layer located adjacent to the plasma membrane (Fig. 3a). In some regions, deposition of pectin can be observed externally to the plasma membrane, becoming incorporated into the intine (Fig. 3c, f-g).

In the pore region, the intine is thickened, exhibiting two distinct zones (Fig. 3f). The inner layer of the intine is less electron-dense than the outer one and contains numerous channels filled with pectins and other compounds of mixed nature (Fig. 3f). The vegetative cell displays a dense protoplast, rich in large amyloplasts, lipid droplets, and small vacuoles containing a substance of dense appearance (Fig. 3a, d-e).

**Fig. 4:**
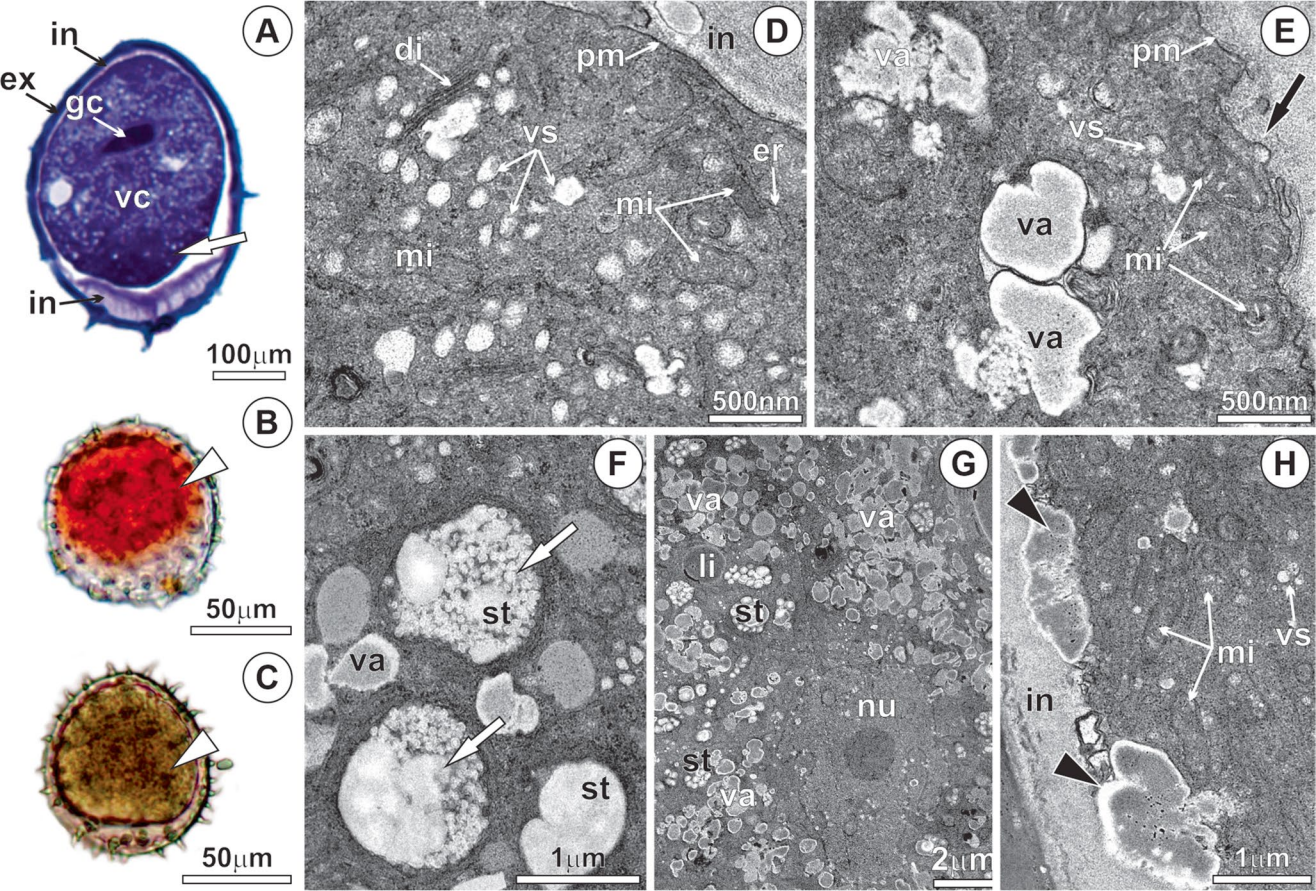
Anatomy and ultrastructure of *Mauritia flexuosa* pollen after the resumption of metabolism - phase II. (**A**) Onset of vegetative cell polarization (white arrow), with mucilage in the thickened intine region (pore region) and mucilage (purple color stained with toluidine blue) accumulation in the vegetative cell protoplast. (**B**) Presence of proteins in the vegetative cell protoplast on the side opposite the pore (white arrowhead), stained with xylidine-ponceau. (**C**) Presence of starch in the vegetative cell protoplast (white arrowhead), stained with Lugol’s reagent. (**D**-**E**) Proliferation of mitochondria, dictyosomes, endoplasmic reticulum, and vesicles in the vegetative cell protoplast after metabolic activation; invagination in the plasma membrane (black arrow). (**F**) Beginning of starch reserve degradation (white arrow). (**G**) Vegetative cell nucleus and numerous vacuoles. (**H**) Accumulation of pectin (black arrowhead) in the intine; mitochondria with well-developed cristae and numerous vesicles. di, dictyosomes; ex, exine; in, intine; li, lipids; mi, mitochondria; pm, plasma membrane; st, starch; va, vacuoles vs; vesicles

In Phase II, metabolic reactivation occurs following pollen rehydration. Polarization of the vegetative cell is evident, characterized by the concentration of cytoplasmic components near the pore region (Fig. 4a) and the onset of protein (Fig. 4b) and starch reserve mobilization (Fig. 4c). This stage is defined by the proliferation of vesicles derived from dictyosomes and the endoplasmic reticulum and organelles (Fig. 4d), as well as by cytoskeletal reorganization in the pore region. Numerous mitochondria with well-developed cristae, dictyosomes, and proliferated endoplasmic reticulum are present in the periphery of the vegetative cell, along with pectin accumulation in the intine (Fig. 4d, h). The vesicles fuse with the plasma membrane and release their contents through a granulocrine-type secretory process (Fig. 4e). In the center of the vegetative cell, the nucleus is large, containing a conspicuous nucleolus (Fig. 4g). The onset of reserve mobilization is evidenced by starch degradation (Fig. 4f) and by vacuole coalescence (Fig. 4g).

**Fig. 5:**
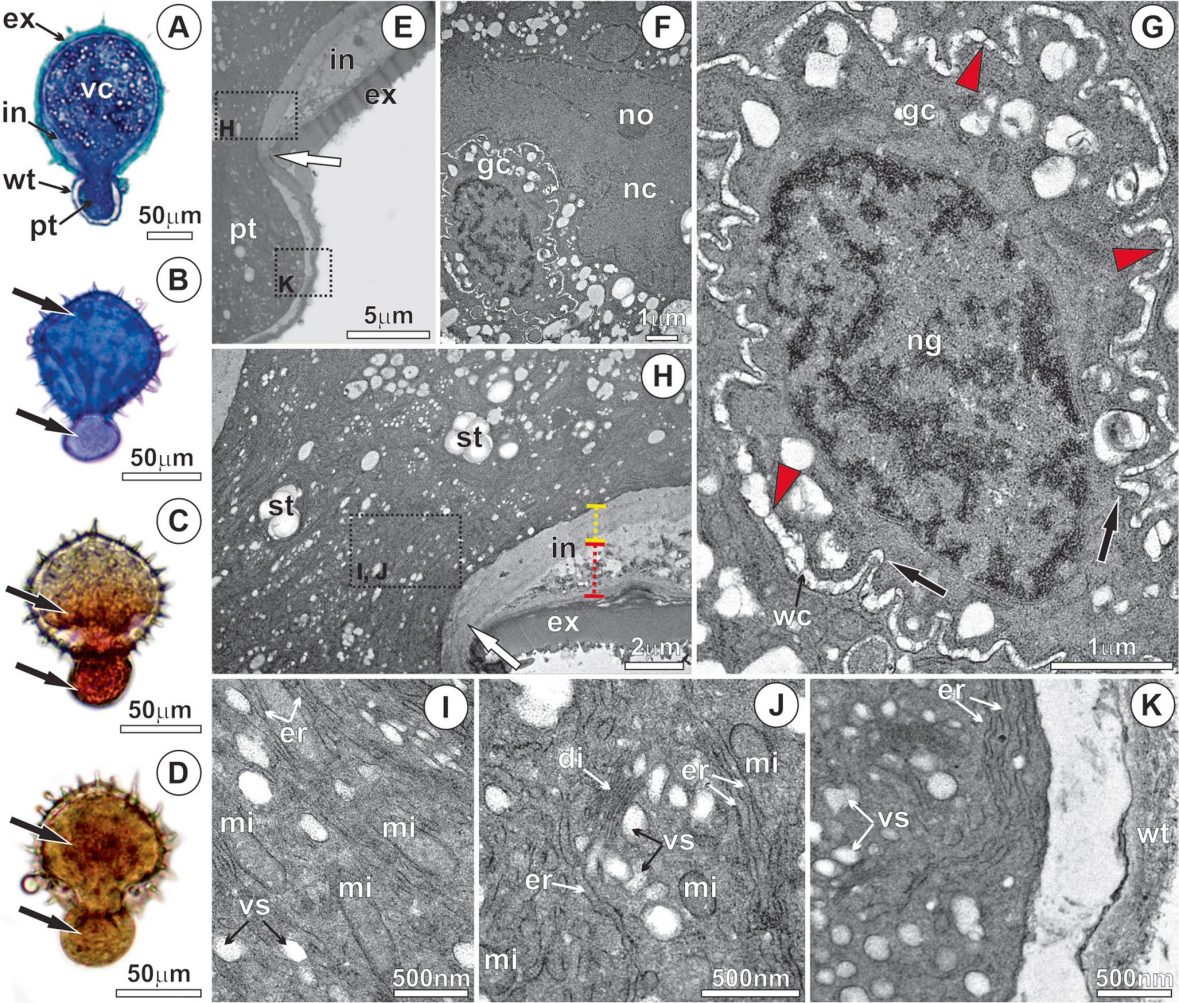
Anatomy and ultrastructure of *Mauritia flexuosa* pollen germination - phase III. (**A**-**B**) Beginning of the germination process, with the projection of the pollen tube wall; mucilage in the vegetative cell protoplast and in the pollen tube (black arrows) stained with toluidine blue. (**C**) Proteins stained with xylidine ponceau; (**D**) Starch, stained with Lugol’s reagent, in the vegetative cell protoplast and in the pollen tube (black arrows). (**E**) Pollen tube protrusion in the pore region, showing the end of the intine (white arrow) and the beginning of pollen tube wall formation. (**F**) Generative cell and vegetative cell nucleus in proximity. (**G**) Undulations in the cell wall of the generative cell (black arrows) and plasmodesmata (red arrowheads) connecting the protoplasts of the vegetative and generative cells. (**H**) Pollen tube protrusion with redirection of the protoplast toward the apical zone of the tube and onset of tube wall formation (white arrow). (**I**) Proliferation and redirection toward the apical zone of the pollen tube of the endoplasmic reticulum and mitochondria with well-developed cristae. (**J**-**K**) Dictyosomes releasing vesicles and secretion in the apical zone of the tube for continuous synthesis and elongation. di, dictyosomes; er, endoplasmic reticulum; ex, exine; gc, generative cell; in, intine; mi, mitochondria; ng, generative cell nucleus; nc, vegetative cell nucleus; pt, pollen tube; st, starch; va, vacuoles; vs, vesicles; wc, generative cell wall; wt, wall pollen tube

In Phase III, pollen tube protrusion occurs, and most of the cellular components are displaced toward its apical portion (Fig. 5a). The nucleus of the vegetative cell is large, with invaginations in the nuclear envelope and a conspicuous nucleolus (Fig. 5f). The generative cell is located adjacent to the vegetative cell nucleus and possesses a thin, sinuous cell wall containing numerous plasmodesmata (Fig. 5f). The generative cell protoplast is poor in organelles and storage substances but contains a large nucleus with condensed chromatin (Fig. 5g). The newly synthesized wall of the pollen tube is continuous with the inner layer of the intine, which projects outward as the vegetative cell elongates through the pore (Fig. 5a, h). In this region, a high concentration of vesicles, endoplasmic reticulum, dictyosomes, and mitochondria is observed (Fig. 5h). Particularly at the apical region of the elongating pollen tube, these organelles proliferate and are involved in the continuous synthesis of the cell wall, which exhibits a loosely organized structure (Fig. 5e, k).

**Fig. 6:**
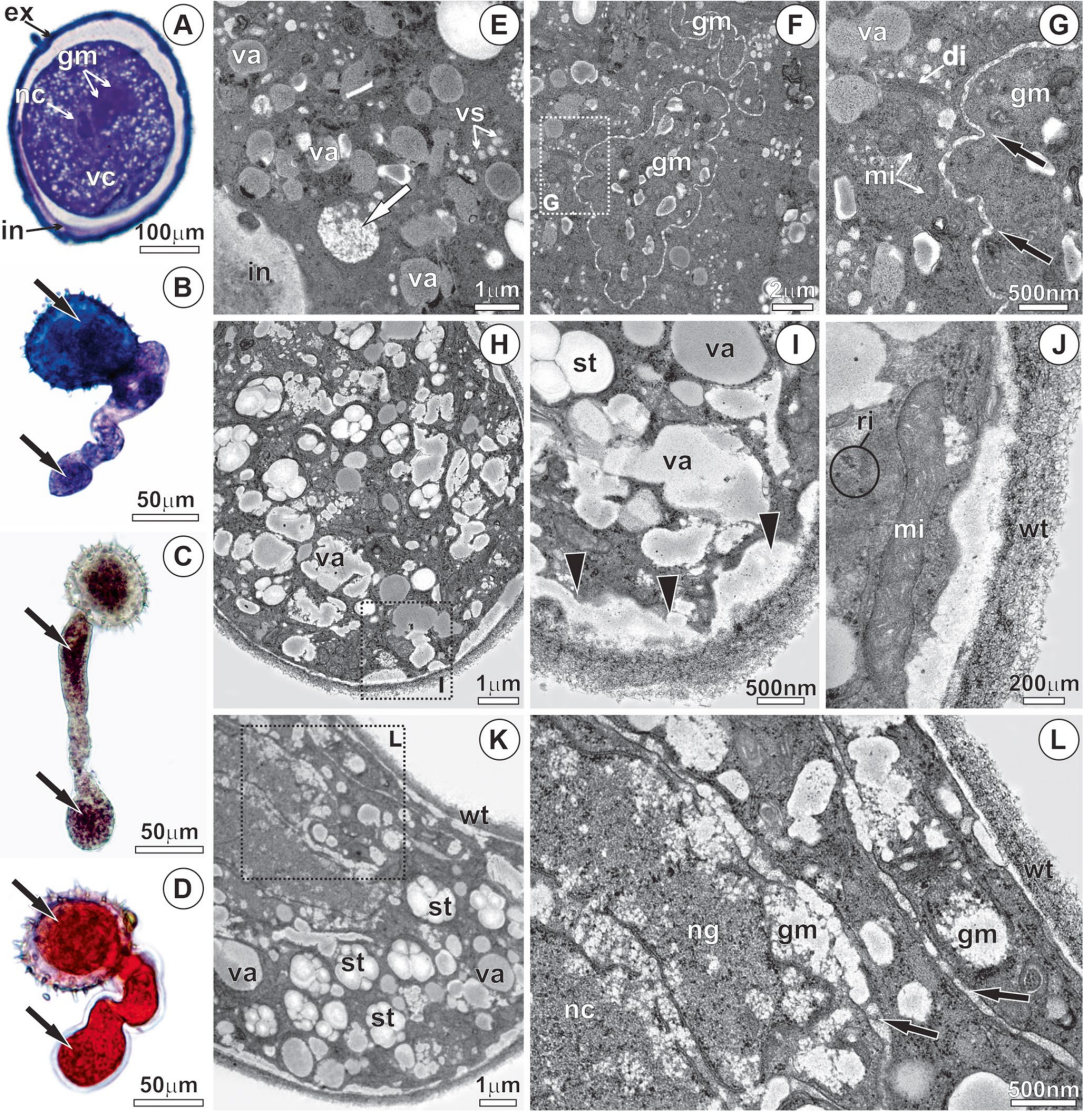
Anatomy and ultrastructure of *Mauritia flexuosa* pollen after pollen tube elongation - phase IV. (**A**) Formation of the microgametes after mitosis. (**B**-**D**) Elongation of the pollen tube with continuous projection of the wall. (**B**) Mucilage, (**C**) Starch, and (**D**) Proteins in the vegetative cell protoplast and in the apical zone of the tube (black arrows). (**E**) Starch degradation (white arrow) and proliferation of vesicles in the periphery of the vegetative cell. (**F**-**G**) Post-Mitosis II, resulting in two microgametes. Small vacuoles containing mucilage, mitochondria (white arrow), and dictyosomes surrounding the microgametes; reduction in the undulation of the microgamete cell wall (black arrows). (**H**-**J**) Apical zone of the pollen tube. (**K**-**L**) Microgametes and vegetative cell nucleus in the apical zone of the pollen tube and continuous synthesis of compounds, with granulocrine secretion for ongoing pollen tube formation. di, dictyosomes; er, endoplasmic reticulum; ex, exine; gm, microgametes; in, intine; mi, mitochondria; pt, pollen tube; st, starch; va, vacuoles; vs, vesicles; wc, generative cell wall

Phase IV is characterized by the elongation of the pollen tube following the second mitosis of the generative cell (Fig. 6a), associated with the accumulation of compounds along its length, while still retaining a portion within the pollen grain (Fig. 6b). After pollen tube emergence, two microgametes are formed, which exhibit a thinner and less sinuous wall compared to their progenitor cell (Fig. 6a, f-g). During pollen tube elongation, pectin accumulates in the apical zone within numerous vacuoles (Fig. 6b, h-i). The elongation of the pollen tube results from intense secretory activity in the apical region, with the release of substances into the periplasmic space and the formation of the wall (Fig. 6h). This region contains numerous vacuoles, mitochondria, ribosomes, endoplasmic reticulum, dictyosomes, and vesicles, as well as starch, proteins, and lipids (Fig. 6h). The microgametes are located in the subapical portion of the pollen tube, close to the vegetative cell nucleus (Fig. 6k).

## Discussion

**Fig. 7:**
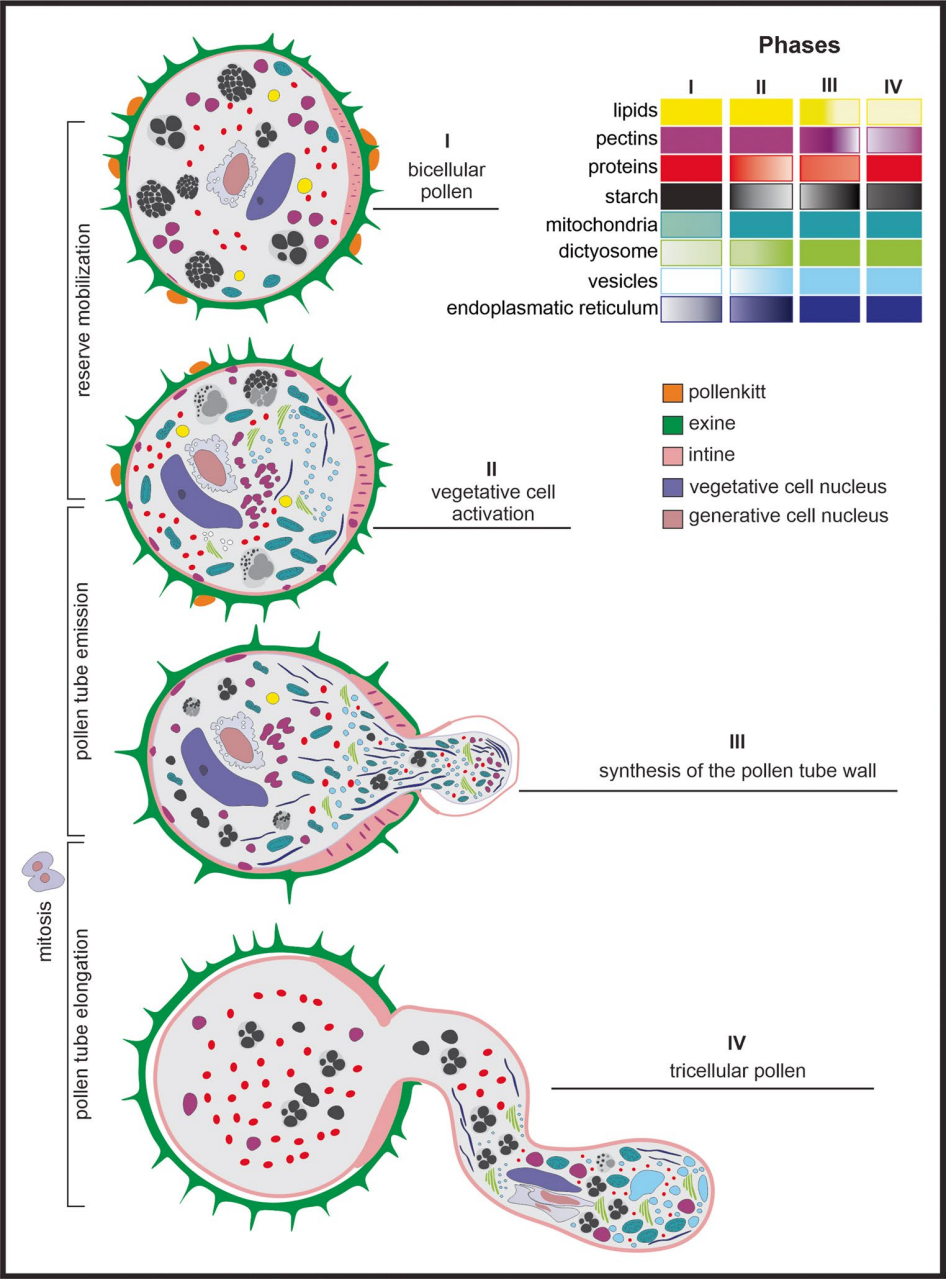
Stages of pollen germination in *Mauritia flexuosa*. The gradient bar represents the dynamics of compound mobilization and organelle distribution across the four germination stages. The light color indicates scarcity, whereas the intense color reflects greater abundance of the compound. Phase I: bicellular pollen with a dense protoplast rich in reserves. Phase II: activation of the vegetative cell after pollen hydration; the onset of reserve mobilization occurs, the organelle population increases, and polarization in the vegetative cell becomes evident. Phase III: pollen tube emergence, with the redirection of the protoplast toward the apical region and continuous synthesis of the tube wall. Phase IV: tricellular pollen; mitosis occurs, leading to the formation of the two microgametes, which are directed toward the subapical portion of the elongating pollen tube; in this phase, there is an accumulation of compounds, especially proteins and starch

This study provides unprecedented information on the cytological aspects of pollen germination in palms. Although Arecaceae holds great ecological and economic importance, such detailed information on pollen germination and pollen tube development was, until now, nonexistent for the family. In *M. flexuosa*, the process occurs rapidly, with remarkable synchrony between the mobilization of reserves and other subcellular changes for pollen tube development, which were characterized in four phases (Fig. 7). The abundance of reserve compounds in the vegetative cell indicates possible autonomy for the rapid activation of germination and the initial growth of the pollen tube. The mixed feature of the intine, especially in the pore region, where it is thickened, provides efficiency for pollen hydration and germination. The early second mitosis, soon after pollen tube emergence, gives rise to microgametes and may be associated with greater speed of fertilization. This aspect is particularly relevant, considering the reproductive biology of the species, which is dioecious, has short-lived flowers, and occurs in environments with supra-annual flowering. These data contribute to broadening the understanding of the reproductive cycle of the species occurring in the veredas and may support domestication and conservation studies.

### Micromorphological, cytochemical, and ultrastructural characteristics of the pollen

The pollen of *M. flexuosa* exhibits structural and functional traits associated with high desiccation resistance, rapid hydration, germination, and microgametes formation, as well as interactions with animals that contribute to cross-pollination. The structure and composition of the sporoderm are noteworthy, with the exine ornamented and composed of phenolic compounds, and the intine of mixed composition, containing abundant pectin, particularly in the pore region. The high concentration of storage compounds in the vegetative cell provides a rapid energy source, linked to the dynamic reorganization of the protoplast for pollen tube emission, followed by the formation of microgametes.

The pollen of *M. flexuosa* was identified as having a monoporate aperture, although sulcate openings are frequently observed. In palms, pollen typically presents a single aperture, with patterns varying among species (Sannier et al. 2006). The monosulcate type is the most common in the Arecaceae and in other monocotyledons (Dahlgren and Clifford 1982). Although the monoporate aperture type is less represented (Harley and Zavada 2000), it has been reported in other, more basal species within the subfamily Calamoideae, such as in the genera *Pogonotium* and *Mauritiella* (Harley and Backer 2001). The monoporate type observed in our study region appears to be a characteristic of local populations of *M. flexuosa*. The polymorphism observed in buriti pollen may be associated with its phenotypic autoplasticity, given the species’ remarkable genetic variability (Collevatti et al. 2024). Despite the difference in aperture type in this species’ pollen, there is still no information on its impact on adaptation or pollen resistance under different environmental conditions. However, aperture size may influence the degree of pollen vulnerability to environmental changes (Edlund et al. 2004).

The exine exhibits a spinulose ornamentation and is predominantly composed of phenolic compounds, in addition to the presence of abundant pollenkitt. This ornamentation pattern is strongly associated with interactions with insects, facilitating cross-pollination. Work conducted by our research group on *M. flexuosa* occurring in semi-arid vereda regions indicated predominantly insect-mediated pollination (data in preparation). This finding is supported by pollen structure and composition, as well as by the pattern of post-pollination events described by Mazzottini-dos-Santos et al. (2025). Our data contrasts with the observations of Rosa and Koptur (2013), who reported anemophily for the species occurring in the Amazon region. The lipid composition of the pollenkitt is also related to the attraction and adhesion to insect bodies (Lersten 2004), as well as promoting attachment to the stigma surface (Edlund et al. 2004; Lin et al. 2013; Adhikari et al. 2020; Mazzottini-dos-Santos et al. 2025). The stigma of the buriti flower bears mucilage-secreting papillae and presents a surface supplied by vascularization, which contributes to pollen grain adhesion and rapid germination. However, this structure exhibits a short period of receptivity and a limited duration of flowers within the inflorescence, as described by Mazzottini-dos-Santos et al. (2025). The micromorphological and physiological characteristics of the pistillate flower and the pollen grain support insect-mediated pollination, thereby ensuring seed production. The detection of phenolic compounds in the exine indicates the presence of sporopollenin, a biopolymer associated with high resistance to UV radiation, microorganisms, and chemical degradation (Cortez et al. 2022).

The composition and characteristics of the intine may influence the efficiency of pollen hydration and the activation of germination. In the aperture region, this layer is notably thickened and contains numerous microchannels impregnated with pectic substances (Fig. 7), as well as proteins and lipids. This thickening pattern is commonly described in pollen of various species, including palms, although detailed data on its ultrastructure and composition remain scarce (Lersten 2004; Sannier et al. 2006).

Upon contact with a hydrated substrate, conspicuous modifications were observed in this region, including the formation of mucilage, a highly hydrophilic substance. Together with subcellular changes, such as protoplast polarization and the onset of reserve mobilization, this region was shown to be crucial for the efficient activation of vegetative cell metabolism, as it facilitates water uptake. Our findings indicate that the pectic composition of the intine favors the expansion of the vegetative cell during the initial phase of pollen tube development and becomes continuous with the newly formed pollen tube wall, similarly to what was characterized by Edlund et al. (2004). In addition, proteins and enzymes present in this region are commonly associated with pollen–pistil recognition and with facilitating pollen tube emergence (Edlund et al. 2004; Lersten 2004).

Pollen is rich in reserve compounds, such as proteins, lipids, pectic substances, and starch. The presence of starch reserves had already been reported for the pollen of this species as the main storage compound (Baker and Baker 1979), highlighting its essential role in providing energy during the initial stages of germination. Our data indicate that pollen germination in *M. flexuosa* occurs rapidly when cultivated in vitro, within approximately 2 h (personal observations). The same pattern was observed during in vivo germination, following pollen contact with the secretory stigma (Mazzottini-dos-Santos et al. 2025). Rapid germination has also been reported in other palms, with little variation (between 2 and 4 h), such as in *Nypa fruticans* (Mantiquilla et al. 2018), *Elaeis guineensis*, and *Elaeis oleifera* (Mosquera et al. 2021). Although pollen is self-sufficient in reserves to trigger rapid germination, we believe that the availability of moisture and sugars in the medium, such as in stigma exudates (Mazzottini-dos-Santos et al. 2025), may be essential for the speed of germination and pollen tube development. In this context, the nature of the stigma plays a decisive role. In buriti, the stigma is wet, secreting exudates rich in water and organic compounds, which promotes rapid pollen hydration and the onset of germination (Mazzottini-dos-Santos et al. 2025). In dry stigmas, pollen hydration occurs in a more restricted manner, but it is still essential for successful germination, as shown in studies with self-incompatible lines of *Arabidopsis*, in which germination takes place within the first 10 min after contact with the stigmatic surface (Rozier et al. 2020).

As highlighted by Sunil Kumar et al. (2011), the basic composition of the culture medium is critical for germination, but specific requirements may vary according to genetic demands. Therefore, future studies are essential to identify the composition of stigma exudates and their role in the nutrition of the growing pollen tube.

### Phases of germination, pattern of reserve mobilization, and subcellular alterations

The mobilization of storage compounds and the dynamics of subcellular alterations in pollen are highly coordinated and complex processes, essential for promoting germination and the elongation of the pollen tube toward the ovule. In this study, four phases of pollen germination in *M. flexuosa * were identified and characterized: (I) immature, bicellular pollen; (II) rehydration with activation of the vegetative cell protoplast, intine thickening, and onset of reserve mobilization; (III) pollen tube emergence with protoplast reorientation; and (IV) second mitosis and pollen tube elongation, with protoplast concentration in the apical region of the tube (Fig. 7).

In phase I, *M. flexuosa* pollen is dispersed with high amount of compounds in the vegetative cell protoplast and with small vacuoles. These features are closely associated with the ability to preserve viability during pollination (Pacini et al. 2006), similarly to the physiological traits of orthodox seeds that maintain desiccation tolerance during dispersal.

Despite its high resistance, the sporoderm exhibits a certain degree of flexibility, allowing pollen grains to adjust their shape and size according to fluctuations in water availability during development and transport. This adaptive mechanism, known as harmomegathy (Wodehouse 1935), ensures that pollen can physiologically respond to environmental variations. The hydration level of pollen at the time of dispersal varies among species, and its viability is preserved by specialized apertures in the pollen wall that enable it to withstand volume changes (Cortez et al. 2022).

Although studies on the dynamics of pollen reserve mobilization are limited in Arecaceae, a similar pattern has been observed during the early germination of *Trachycarpus fortunei* pollen (Guarnieri et al. 2006). One of the first subcellular alterations we observed in buriti pollen was the polarization of the vegetative cell protoplast in the pore region and the onset of the secretory phase. In this process, vesicles derived from dictyosomes fuse with the plasma membrane, characterizing granulocrine secretion (Paiva 2016) and promoting intine thickening, a pattern like that reported in other studies (Krichevsky et al. 2007; Grebnev et al. 2017). Our data indicates that pectin is secreted at the intine extremities and accumulates in the pollen aperture region, increasing its hydrophilic potential and facilitating pollen rehydration upon contact with the stigma. Following starch mobilization, proteins stored in the pollen protoplast begin to be mobilized, followed by lipids, which, although present in lower amounts, may contribute to the stabilization and protection of cellular structures (Shi and Yang 2010).

In this study, in vitro cultivation demonstrated that pollen can absorb nutrients from the culture medium and resynthesizing starch during pollen tube growth, thereby simulating the natural interaction with the stigma. The abundance of compounds available within the tube cell suggests that pollen utilizes its reserves until it establishes interaction with the pistil, ensuring a continuous supply of nutrients and supporting pollen tube growth (Hafidh et al. 2016). Interestingly, studies on *Olea europaea* L. (olive) have shown that the absence of sugars in the culture medium does not affect pollen germination or pollen tube growth rates (Zienkiewicz et al. 2013), indicating that soluble sugars resulting from starch mobilization, together with lipid reserves, may be sufficient to initiate germination (Hernández et al. 2020). Although lipid mobilization was not detected in this phase, he self-sufficiency mechanism of the microgametophyte remains unchanged, supplying energy for the continuous synthesis of the cell wall until the male gametes are delivered to the ovule (Shi and Yang 2010).

In phase III, pollen tube emergence occurs concomitantly with the reorientation of the vegetative cell protoplast toward the apical region, directing its growth exclusively in this direction, as observed and described in the model species *Nicotiana tabacum* and *Arabidopsis thaliana* (Grebnev et al. 2020). At this stage, significant subcellular reorganization takes place, with marked proliferation of organelles such as mitochondria, endoplasmic reticulum, dictyosomes, and secretory vesicles, which are essential for cell wall formation during pollen tube expansion, as suggested by Selles et al. (2018) and Ruan et al. (2021).

Although the generative cell possesses a protoplast poor in organelles and limited reserves, it exhibits essential features for the rapid formation of microgametes. Its large nucleus, with condensed chromatin, indicates readiness for the second mitosis, while intercellular communication with the vegetative cell, mediated by numerous plasmodesmata, ensures functional integration between the two cells. This interaction, described as the male germ unit, facilitates the movement of the microgamete alongside the vegetative cell nucleus (Jiang et al. 2015). In *Nicotiana alata*, this communication is crucial for the coordinated movement of microgametes within the pollen tube, underscoring its importance for successful plant reproduction (Cresti et al. 1987). Although the second mitosis is critical for microgamete formation, studies correlating the speed of this cell division with the total pollen tube germination time are scarce in palms. Only a single investigation provides detailed insights into the metabolic interaction between the vegetative cell protoplast and the microgametes, suggesting it occurs via plasmodesmata (Cresti et al. 1987).

Pollen faces significant challenges, such as maintaining viability, recognizing compatibility, competing for germination, and growing through the transmitting tissue. Simultaneously, there is a race of pollen tubes toward the ovule, guided by attraction signals that select the most suitable genotype (Zhong and Qu 2019). To support this process, the abundance of energetic reserves is essential, particularly when combined with the pollen’s ability to absorb and utilize resources provided by the pistil (Hafidh et al. 2016; Adhikari et al. 2020). In buriti pollen, the abundant protein reserves may be directly related to enzyme synthesis and stigma recognition. Studies on pistillate flowers indicate that the stigma and the papillae of the style channel secrete protein-rich mucilage (Mazzottini-dos-Santos et al. 2025), which may play an essential role in compatibility recognition, a process that depends on intense enzymatic activity (Mizuta and Higashiyama 2018; Adhikari et al. 2020). Such investigations have focused primarily on model species, such as whereas the specific characteristics of this interaction in Arecaceae remain poorly explored and require further attention.

In phase IV, immediately after pollen tube emergence, the second mitosis occurs, producing two microgametes, a process essential for successful fertilization. The synchrony between pollen germination and rapid microgamete formation can be considered a key factor for the reproductive success of *M. flexuosa* in the vereda environment of the semi-arid region. Histochemical assays reveal that the pollen tube wall, with a composition like that of the intine, exhibits the plasticity necessary to penetrate the stigma papillae (Mazzottini-dos-Santos et al. 2025). As the pollen tube grows, its wall becomes thicker and more resistant, providing the structural support required for continued expansion, which occurs exclusively in the apical region (Baillie et al. 2024). In this ecosystem, the dioecious species exhibits supra-annual flowering (Ávila et al. 2023) and flowers with a short anthesis and receptivity period (Mazzottini-dos-Santos et al. 2025). Therefore, rapid pollen tube growth and timely transport of microgametes may ensure higher seed production. This set of conditions imposes considerable selective pressure, as pollination success must be maximized within a relatively short period to ensure species continuity (Ávila et al. 2023). In this context, it is noteworthy that buriti is native to the Amazon, and together with *Mauritiella armata*, has successfully established in the Cerrado, highlighting the efficiency of its reproductive adaptations for species perpetuation in this environment.

## Conclusion

The pollen of *M. flexuosa* exhibits an exine ornamented with sharp spicules and abundant pollenkitt, contributing to efficient pollination. The intine has a mixed composition and is thickened in the aperture region, where it forms mucilage, which is associated with efficient pollen rehydration and rapid germination. The vegetative cell is rich in storage compounds, such as starch, proteins, and lipids, allowing it to sustain metabolic activation for pollen germination and the initial growth of the pollen tube. The generative cell does not possess a dense protoplast, but cytoplasmic connections with the vegetative cell support the dynamics of microgamete formation. Based on subcellular alterations, buriti pollen germination was characterized in four phases: (I) immature bicellular pollen, (II) rehydration with metabolic activation and onset of reserve mobilization, (III) pollen tube emergence with protoplast reorientation, and (IV) mitosis for microgamete formation and pollen tube elongation. Early mitosis for microgamete formation and rapid pollen tube development was recorded in buriti pollen grains. These characteristics may reflect a strategy compatible with the reproductive phenology of the species, which exhibits a short period of stigma receptivity and limited flower longevity. The synchrony between reserve mobilization and subcellular alterations ensures efficient pollen tube development, with continuous cell wall synthesis in the apical region. Starch is the first reserve mobilized, providing initial energy for germination, followed by proteins and lipids. As the pollen tube grows and contacts the substrate, new synthesis of compounds occurs. The cytological features and germination dynamics observed in this study may support future research aimed at the domestication and conservation of buriti, as well as enhancing understanding of its adaptive mechanisms in semi-arid environments. Although this study represents an advance in the understanding of buriti reproduction, there remains a substantial knowledge gap regarding the ontogeny of the megagametophyte and its synchrony with microgametophyte formation. Particularly considering dioecy and its phenological particularities in the semiarid environment, there is a clear need for further studies to better understand the reproductive success of the species.

## Data Availability

The data that support the findings of this study are available from the corresponding author upon reasonable request.
